# PRELIMINARY SOMMA DATA: COGNITIVE AND PHYSICAL FUNCTION RELATIONSHIPS IN OLDER ADULTS

**DOI:** 10.1093/geroni/igac059.834

**Published:** 2022-12-20

**Authors:** Stephen Kritchevsky, Anne Newman, Peggy Cawthon, Theresa Mau, Russell T Hepple, Paul Coen, Bret Goodpaster, Steven Cummings

**Affiliations:** Wake Forest School of Medicine, Winston-Salem, North Carolina, United States; University of Pittsburgh, Pittsburgh, Pennsylvania, United States; California Pacific Medical Center Research Institute, San Francisco, California, United States; California Pacific Medical Center, San Francisco, California, United States; University of Florida, Gainesville, Florida, United States; AdventHealth, Orlando, Florida, United States; AdventHealth, Orlando, Florida, United States; San Francisco Coordinating Center, San Francisco, California, United States

## Abstract

We used canonical correlation (n=424) to examine the relationships between a set of physical function measures (400m usual pace, chair stands/sec, 4m walk pace, standing balance times, VO2 peak, muscle power, four-square step test (FSST) time, stair climb time and stair climb power) and a set of cognitive measures (Trail Making Test B (sec), Digit Symbol Substitution Test (DSST), the Montreal Cognitive Assessment, and California Verbal Learning Test). Canonical correlation derives synthetic variables comprised of linear combinations within each set of variables (physical and cognitive) that maximize the correlations between synthetic variables. The FSST was most strongly correlated with the cognitive synthetic variable (- 0.42). The DSST score was the cognitive measure most strongly correlated with the physical synthetic variable (0.48). It is notable that only the timed cognitive and physical tests are inter-associated

